# Real-world clinical experience with upadacitinib in a cohort of Italian patients with axial spondyloarthritis

**DOI:** 10.3389/fphar.2026.1757110

**Published:** 2026-04-29

**Authors:** Roberta Ramonda, Mariagrazia Lorenzin, Antonio Carletto, Maria Sole Chimenti, Maria Manara, Giacomo Cozzi, Alen Zabotti, Roberta Foti, Antonio Carriero, Ariela Hoxha, Carlo Selmi, Bernd Raffeiner, Alessandro Giollo, Alberto Cauli, Carlo Salvarani, Fabiola Atzeni, Maurizio Rossini, Angelo Fassio, Francesca Nava, Valentino Paci, Gianluca Moroncini, Simona Buonanno, Bruno Frediani, Michele Maria Luchetti Gentiloni, Stefano Gentileschi

**Affiliations:** 1 Department of Medicine DIMED, Rheumatology Unit, Padova University Hospital, Padova, Italy; 2 Department of Surgery, Oncology and Gastroenterology (DiSCOG), Padova University Hospital, Padova, Italy; 3 Department of Medicine, Rheumatology Unit, AOUI University of Verona, Verona, Italy; 4 Department of Systems Medicine, Rheumatology, Allergology and Clinical Immunology, University of Rome Tor Vergata, Rome, Italy; 5 Department of Rheumatology, ASST Gaetano Pini-CTO, Milan, Italy; 6 Department of Medicine, Rheumatology Division, University of Udine and Azienda Sanitaria Universitaria del Friuli Centrale, Udine, Italy; 7 Rheumatology Division, AOU Policlinico “G. Rodolico” San Marco, Catania, Italy; 8 Rheumatology Department of Lucania, San Carlo Hospital of Potenza, Potenza, Italy; 9 Department of Medicine-DIMED, Thrombotic and Haemorrhagic Diseases Unit, Internal Medicine, Padova University Hospital, Padova, Italy; 10 Department of Biomedical Sciences, Humanitas University, Milan, Italy; 11 Department of Rheumatology and Clinical Immunology, IRCCS Humanitas Research Hospital, Milan, Italy; 12 Department of Rheumatology, Central Hospital of Bolzano (SABES-ASDAA), Teaching Hospital of Paracelsus Medical University (PMU), Bolzano, Italy; 13 Department of Medical Sciences, Rheumatology Unit, AOU and University of Cagliari, Monserrato, Italy; 14 Department of Rheumatology, University Hospital of Modena and Reggio Emilia, Reggio Emilia, Italy; 15 Rheumatology Unit, University of Messina, Messina, Italy; 16 Department of Clinical and Molecular Sciences, University Hospital of the Marche Region, Ancona, Italy; 17 Department of Medical Sciences, Surgery and Neuroscience, Rheumatology Unit, University of Siena, Siena University Hospital, Siena, Italy

**Keywords:** axial spondylarthritis, biologics, drug retention rate, Janus kinase inhibitors, real-world evidence (RWE), upadacitinib

## Abstract

**Background:**

There is currently scarce data on the real-world effectiveness and safety of upadacitinib in patients with axial spondyloarthritis (axSpA). This study evaluated clinical outcomes and drug retention rate (DRR) at 6, 12, and 24 months in patients initiating upadacitinib for axSpA.

**Methods:**

This prospective, observational study enrolled consecutive patients with radiographic axSpA (r-axSpA) or non-radiographic axSpA (nr-axSpA) initiating upadacitinib between December 2022 and January 2025 at 16 Italian centres. The primary endpoints were treatment effectiveness—evaluated using clinimetric indices, including the Bath Ankylosing Spondylitis Disease Activity Index (BASDAI) and Ankylosing Spondylitis Disease Activity Score (ASDAS) — safety and DRR at 6, 12, and 24 months.

**Results:**

Overall, 203 patients were analysed (48% male; median age at diagnosis 44 years; 22.7% HLA-B27 positive; 71% with nr-axSpA). Sixty-three patients completed the 24-month follow-up. Upadacitinib demonstrated a statistically significant improvement in effectiveness outcomes at 6 months vs. baseline, which was sustained at 12 and 24 months, regardless of previous biologic therapy lines, axSpA subtype, or sex. Furthermore, DRR was 92%, 80%, and 55% at six, 12, and 24 months, respectively. Forty patients discontinued upadacitinib, including 22 for insufficient effectiveness and two for AEs. Twenty-two patients developed infections.

**Conclusion:**

Upadacitinib demonstrated a favourable effectiveness profile in the evaluable axSpA population with sustained remission and high treatment retention over a two-year follow-up. Within the limitations inherent to this observational study, the tolerability profile of upadacitinib was generally consistent with that reported in randomized controlled trials, and no new safety signals were identified. Overall, these findings support the effectiveness of Janus kinase inhibitors in the treatment of patients with axSpA.

## Introduction

Axial spondyloarthritis (axSpA) is a chronic inflammatory disease of the axial skeleton causing persistent back pain (Sieper and Poddubnyy, 2017). Before magnetic resonance imaging (MRI), ankylosing spondylitis was diagnosed when sacroiliac joint (SIJ) damage appeared on x-ray, but MRI can detect inflammation before visible structural damage (Robinson et al., 2021). This led to classifying axSpA as radiographic (r-axSpA) or non-radiographic (nr-axSpA) according to the presence or absence of X-ray findings (i.e., joint space narrowing, erosions, ankylosis, sclerosis) (Robinson et al., 2021). Besides spine and SIJ involvement, patients may develop peripheral arthritis, enthesitis, dactylitis, or extra-articular manifestations like psoriasis, uveitis, or inflammatory bowel disease (Sieper and Poddubnyy, 2017; Robinson et al., 2021).

In Italy, the prevalence of ankylosing spondylitis (now termed r-axSpA) is 88.3 per 100,000 adults (Perrone et al., 2022), though it may be underdiagnosed. A study in Northern Italy found undiagnosed axSpA in 22% of patients being investigated for nongranulomatous anterior uveitis (Bolletta et al., 2022). There is a strong genetic component to axSpA, with an estimated 90% heritability and high prevalence of human leukocyte antigen (HLA) variants, particularly HLA-B27 (Sieper and Poddubnyy, 2017).

Guidelines from the Assessment of Spondyloarthritis International Society (ASAS) and the European Alliance of Associations for Rheumatology (EULAR) (Ramiro et al., 2023) and the Italian Society for Rheumatology (Manara et al., 2021) recommend initiating treatment in axSpA patients with non-steroidal anti-inflammatory drugs (NSAIDs). Biologics or Janus kinase inhibitors (JAKis) are advised if pain persists (Ramiro et al., 2023); conventional synthetic DMARDs (csDMARDs) are reserved for peripheral arthritis, not axial disease (Ramiro et al., 2023; Manara et al., 2021). Biologics against tumour necrosis factor (TNF)α or interleukin (IL)-17 (Ramiro et al., 2023; Manara et al., 2021) require injections, achieving ASAS40 responses in ∼50–65% of patients (Navarro-Compan et al., 2017), whereas oral JAKis block multiple cytokines, including IL-23 and downstream IL-17 (Ahmed et al., 2024; Nash et al., 2021).

Upadacitinib is a JAKi approved in Europe since 2019 for axSpA and other immune-mediated diseases, including psoriatic arthritis, ulcerative colitis, Crohn’s disease, giant cell arteritis, and atopic dermatitis (European Medicines Agency, 2025). The SELECT-AXIS 1 and 2 trials demonstrated its efficacy and safety in axSpA, including patients with inadequate responses to biologics (Baraliakos et al., 2024; van der Heijde et al., 2019). Moreover, as randomized controlled trials (RCTs) involve selected populations, real-world evidence is increasingly recognized as essential to complement RCT findings in heterogeneous patient populations (Dreyer, 2022; Eichler et al., 2021).

This ongoing prospective observational study endeavors to investigate the real-world effectiveness and safety of upadacitinib, as well as the drug retention rate (DRR), in real-world Italian rheumatology evidence. Here, we present data from a cohort of patients affected by axSpA followed for 24 months.

## Methods

### Study design

This prospective observational study began in December 2022 across 16 Italian centers involving patients with axSpA. Ethics approval (no. 3572/AO/15) was obtained and acknowledged by all centers. All participants gave written informed consent. The study was conducted in compliance with Italian regulations, the principles of the Declaration of Helsinki (2013), and ICH Good Clinical Practice guidelines.

### Patients and treatment

Patients were eligible for inclusion if they had been diagnosed with axSpA according to ASAS criteria (Rudwaleit et al., 2009), had moderate to severe disease according to the EULAR or ACR guidelines (Ramiro et al., 2023; Ward et al., 2019), and had chosen to initiate upadacitinib after discussion of treatment options with their physician. All patients who met these criteria were willing to participate and provided written informed consent were included (all-comers); the only exclusion criterion was use of upadacitinib for an indication other than moderate to severe axSpA. The recommended dose of upadacitinib for axSpA is 15 mg once daily (European Medicines Agency, 2025).

### Assessments and follow-up

All patients underwent comprehensive medical and physical examinations conducted by rheumatologists. Demographic data (age, sex, BMI) and disease-related information (back pain onset and duration, age at diagnosis, history/presence of arthritis, enthesitis, dactylitis, extra-articular manifestations, response to NSAIDs, and family history of SpA) were collected. Laboratory tests included CRP, ESR, and HLA-B27, with SIJ X-ray and MRI performed when clinically indicated. Data on prior or ongoing treatments (csDMARDs, NSAIDs, glucocorticosteroids, and biologics) and on comorbidities or concomitant therapies were also recorded. Baseline information was obtained through interviews and medical records. Treatment response to upadacitinib was evaluated at 6, 12, and 24 months using VAS-Pain, VAS-GH, VAS-PA, ASDAS, BASFI, BASDAI, BASMI, and LEI. We reassessed ESR and CRP at each timepoint. Low disease activity was defined as BASDAI <4 or ASDAS <2.1, and very low as BASDAI <2 or ASDAS <1.3. All adverse events, infections, and hospitalizations were documented.

### Endpoints

Our primary endpoints were effectiveness, safety, and drug retention rate (DRR) at 24 months. We calculated DRR from the first to last dose of upadacitinib, and reasons for discontinuation were recorded. Effectiveness was also assessed at 6 and 12 months using VAS-Pain, VAS-GH, VAS-PA, BASFI, BASDAI, BASMI, LEI, ASDAS, ESR, and CRP. The proportions of patients achieving low disease activity (LDA) or very low disease activity (VLDA) at 6, 12, and 24 months were calculated. Safety analyses included adverse events, infections, hospitalizations, and treatment discontinuations due to AEs. Secondary objectives evaluated predictors of LDA/VLDA and DRR, including age at symptom onset, male sex, HLA-B27 positivity, baseline sacroiliitis on MRI, elevated CRP, baseline BASDAI >4, and prior therapy lines. The impact of comorbidities, such as cardiovascular disease and diabetes, on DRR was also assessed to identify factors influencing treatment, persistence and outcomes.

### Statistical analysis

Data were analyzed by descriptive analysis, with categorical variables expressed as frequencies and percentages and continuous variables as median and range. The primary endpoint analyses were undertaken in the evaluable population, i.e., all patients who were still receiving upadacitinib at the 24-month follow-up and had at least one evaluable parameter at each follow-up. Analyses were also conducted in effectiveness population, i.e., all patients who had at least one evaluable parameter at any timepoint.

The comparison between groups was carried out with the Chi-Square or Fisher’s Exact test for categorical variables while non-parametric exact Mann-Whitney U-tests was used for continuous variables. The McNemar test was used for paired categorical variables to assess changes in proportions over time. Differences over time for continuous variables were evaluated using the Friedman test and Wilcoxon signed-rank test, when appropriate.

Non-parametric tests (Friedman test, Wilcoxon signed-rank test, and Mann–Whitney U test) were selected due to the non-normal distribution of the data and/or the small sample size. No imputation was made for missing data, and no adjustment for multiplicity was made to adjust for a type I error in secondary endpoints. A p-value of 0.05 was considered to be statistically significant.

Factors evaluated for their impact on LDA or VLDA at 6, 12 and 24 months were previous lines of treatment (≤2 or >2), axSpA subtype (radiographic or non-radiographic) and sex (male or female). These variables were also considered in the analysis of factors that influence DRR, as well as comorbidities (yes or no), age (≥50 or <50 years), disease duration (≤5 or >5 years), HLA-B27 status (positive or negative), sacroiliitis on MRI (present or absent), smoking status (yes or no) and peripheral involvement (with or without peripheral arthritis). Logistic regression models were applied to evaluate the association between predictors and binary outcomes, while Cox proportional hazards regression models were used for time-to-event analyses.

Odds ratios (ORs) and hazard ratios (HRs) with corresponding 95% confidence intervals were estimated for each factor using logistic regression and Cox univariate models, respectively. Univariate regression models were used for exploratory association analyses.

All reported P values are based two-sided tests, and a P value of less than 0.05 was considered to indicate statistical significance. SPSS (version 30.0, SPSS Inc., Chicago, Illinois, USA) statistical program was used for all analyses.

## Results

### Patients

The cutoff date for data analysis was January 2025. At this timepoint, 203 patients had been enrolled: 106 females and 97 males, aged 20 to 80 (median 53) years (Table 1). Most patients (n = 144 [70.9%]) had nr-axSpA, were negative for HLA-B27 (n = 152 [74.9%]), and had sacroiliitis on MRI (n = 175 [86.2%]). Patients had been diagnosed 10 years previously and receiving treatment for a median 13 months. Most patients were prescribed upadacitinib as third-line or later treatment. Before initiating upadacitinib, 57% (116/203) of patients had been treated with at least one conventional disease-modifying antirheumatic drugs (cDMARDs), and 90.1% (183/203) had prior exposure to biological disease-modifying antirheumatic drugs (bDMARDs).

**TABLE 1:** Baseline patient demographic and clinical characteristics.

Characteristic	Upadacitinib (n = 203)^a^	Lines of prior biologic therapy	
		<2	≥2
Age, years, median (range)	53 (20–80)	54 (21–71)	53 (20–80)
Sex, n (%)	​	​	​
Male	97 (47.8)	27 (52.9)	79 (52.0)
Female	106 (52.2)	24 (47.1)	73 (48.0)
Region of residence, n (%)	​	​	​
Northern Italy	83 (40.9)	16 (31.4)	67 (44.1)
Central Italy	106 (52.2)	32 (62.7)	74 (48.7)
Southern Italy	14 (6.9)	3 (5.9)	11 (7.2)
Body mass index, kg/m^2^ median (range)	25.4 (17–46)	24.7 (17–40)	25.6 (17–46)
Age at axSpA diagnosis, years, median (range)	44 (14–73)^b^	49 (14–68)	42 (15–73)
Disease duration, years median (range)	10.0 (1–56)^c^	7 (1–27)	11 (1–56)
Smoking status, n (%)	​	​	​
Current	48/197 (23.6)	12 (23.5.)	36 (24.7)
Non	149/197 (73.4)	39 (76.5)	110 (75.3)
Family history^d^, n (%)	49/201 (24.1)	9 (17.6)	40 (26.7)
AxSpA subtype, n (%)	​	​	​
Non-radiographic	144/202 (70.9)	40 (78.4)	104 (68.9)
Radiographic	58/202 (28.6)	11 (21.6)	47 (31.)
Positive for HLA-B27, n (%)	46/198 (22.7)	8/51 (15.7)	38/147 (25.9)
Northern Italy	30/82 (36.6)	5/16 (31.2)	25/66 (37.9)
Central-Southern Italy	16/116 (13.8)	3/35 (8.6)	13/81 (16.0)
Sacroiliitis present on baseline MRI, n (%)	175/199 (86.2)	47/51 (92.2)	128/148 (86.5)
Psoriasis, n (%)	67/201 (33.0)	13/51 (25.5)	54/150 (36.0)
Nail involvement, n (%)	25/199 (12.3)	8/51 (15.7)	17/148 (11.5)
Peripheral arthritis, n (%)	136/201 (67.0)	35/51 (68.6)	101/150 (67.3)
Enthesitis, n (%)	96/197 (47.3)	24/51 (47.1)	72/146 (49.3)
Dactylitis, n (%)	22/201 (10.8)	7/51 (13.7)	15/150 (10.0)
IBD, n (%)	39 (19.2)	6 (11.8)	33 (21.7)
Uveitis, n (%)	14/201 (6.9)	2 (3.9)	12 (8.0)
Previous csDMARDs, n (%)	116 (57.1)	25/51 (49.0)	91/152 (59.9)
Previous lines of biologic therapy, n (%)			
0	14 (6.9)	​	​
1	37 (18.2)	​	​
2	61 (30.0)	​	​
3	48 (23.6)	​	​
4	24 (11.8)	​	​
≥5	19 (9.4)	​	​
Line of treatment for upadacitinib, median (range)	3 (1–12)	​	​

^a^ Some datapoints were missing for some patients, as indicated, but the denominator for all percentage calculations was 203.

^b^ n = 199.

^c^ n = 194.

^d^ family history for any inflammatory arthritis.

axSpA, axial spondyloarthritis; csDMARDs, conventional synthetic disease-modifying antirheumatic drugs; HLA, human leukocyte antigen; IBD, inflammatory bowel disease; MRI, magnetic resonance imaging.

Extra-articular manifestations were present in some patients: peripheral arthritis (n = 136 [67.0%]), enthesitis (n = 96 [47.3%]), psoriasis (n = 67 [33.0%]), inflammatory bowel disease (n = 39 [19.2%]), nail involvement (n = 25 [12.3%]), dactylitis (n = 22 [10.8%]), and uveitis (n = 14 [6.9%]) The most common comorbidities were hypertension, dyslipidaemia and fibromyalgia, but these were present in ≤25% of patients (Supplementary Table S1).

**FIGURE 1:**
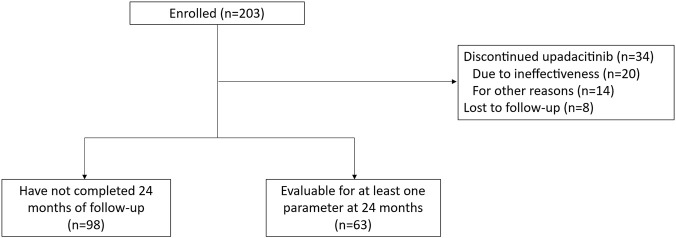
Patient flow chart.

Among the 203 patients, 34 permanently discontinued treatment with upadacitinib (2 additional patients stopped temporarily but restarted), 8 were lost to follow-up and 98 had not completed 24 months of follow-up. The evaluable population consisted of 63 patients who had at least one evaluable parameter at baseline, 6, 12 and 24 months of treatment (Figure 1). Patients in the evaluable population were generally similar to those in the effectiveness population, except that they had a significantly higher incidence of peripheral arthritis (83.6% vs. 60.7%; p = 0.001), enthesitis (61.0% vs. 43.5%; p = 0.024), hypertension (33.3% vs. 18.8%; p = 0.029), and cardiovascular disease (7.7% vs. 0%; p = 0.005) and significantly lower incidence of uveitis (1.6% vs. 9.3%; p = 0.05) (Supplementary Table S2).

### Effectiveness and DRR

At 24 months, all effectiveness parameters showed statistically significant improvements compared with baseline in the evaluable population (n = 63; p < 0.0001) (Table 2), with reductions observed at all assessed timepoints. Comparable improvements were also seen in the effectiveness population at each timepoint (Supplementary Table S3).

**TABLE 2:** Effectiveness parameters over time in the evaluable population (patients with continuous data over 24 months), as median (range).

Parameter	N	Baseline	6 months	12 months	24 months	P-value
VAS-pain score	62	8 (2–10)	5 (1–10)	4 (0–8)	3 (0–9)	<0.0001
VAS-GH score	56	7 (2–10)	5 (0–10)	4 (0–7)	2.5 (0–9)	<0.0001
VAS-PA score	56	7 (1–10)	4 (0–7)	2 (0–7)	1 (0–8)	<0.0001
BASMI score	15	0 (0–5)	0 (0–4)	0 (0–4)	0 (0–2)	<0.0001
LEI score	50	0.5 (0–6)	0 (0–4)	0 (0–2)	0 (0–2)	<0.0001
HAQ score	52	0.875 (0–7)	0.5 (0–6)	0.25 (0–7)	0.25 (0–7)	<0.0001
BASDAI score	62	5.9 (0–9.25)	4.0 (0–7.5)	2.1 (0–6.8)	1.95 (0–6.4)	<0.0001
BASFI score	25	5.3 (0–9.2)	1.0 (0–4.2)	1.3 (0–4.6)	1.0 (0–5.0)	<0.0001
ASDAS score	61	2.8 (0.9–5.0)	1.7 (0.5–4.1)	1.3 (0–3.75)	1.1 (0–3.3)	<0.0001
ESR, mm/h	59	11 (0–96)	5 (0–31)	6 (0–54)	6 (0–56)	<0.0001
CRP, mg/dL	63	1.0 (1.0–59.7)	1.0 (1.0–13.0)	1.0 (1.0–25.0)	0.5 (0–7.4)	<0.0001

ASDAS, ankylosing spondylitis disease activity score; BASDAI, bath ankylosing spondylitis disease activity index; BASFI, bath ankylosing spondylitis functional index; BASMI, bath ankylosing spondylitis metrology index; CRP, C-reactive protein; ESR, erythrocyte sedimentation rate; GH, global health; HAQ, health assessment questionnaire; LEI, leeds enthesitis index; PA, physician’s assessment; VAS, visual analogue scale.

**FIGURE 2:**
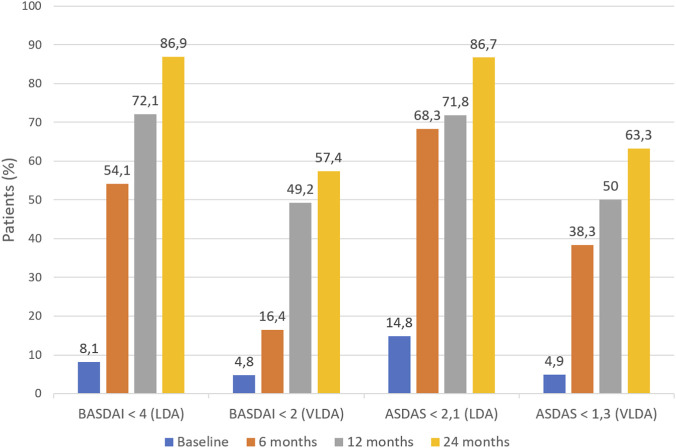
The proportion of patients in the evaluable population who achieved low disease activity (LDA) or very low disease activity (VLDA) at 6, 12 and 24 months using the Bath Ankylosing Spondylitis Disease Activity Index (BASDAI) or the Ankylosing Spondylitis Disease Activity Score (ASDAS). †p < 0.001 vs. preceding timepoint; *p < 0.05 vs. preceding timepoint. n = 61 for most comparisons, but n = 60 for ASDAS<2.1 and ASDAS<1.3 for comparison of baseline vs. 6 months, and 6 months vs. 12 months.

Figure 2 shows the proportion of patients in the evaluable population who achieved BASDAI-LDA, ASDAS-LDA, BASDAI-VLDA and ASDAS-VLDA. Compared with baseline, there was a significant increase in the proportion of patients achieving BASDAI-LDA (p < 0.0001), BASDAI-VLDA (p = 0.039), ASDAS-LDA (p < 0.0001) and ASDAS-VLDA (p < 0.0001) at 6 months. At month 12 vs. month 6, there was a further significant increase in the proportion of patients achieving BASDAI-LDA (p = 0.019) and BASDAI-VLDA (p < 0.0001); and similarly in the proportion of patients achieving BASDAI-LDA (p = 0.012) and ASDAS-LDA (p = 0.021) at 24 months vs. 12 months. Similar trends were seen in the effectiveness population (Supplementary Figure S1).

The rates of LDA and VLDA at 6, 12 and 24 months were generally similar in subgroups of patients stratified by prior lines of biologic therapy, sex and type of axSpA (Table 3), with a few notable exceptions: females had significantly higher rates of LDA at 12 months compared with males (p = 0.018) and patients with nr-axSpA had significantly higher rates of VLDA at 24 months compared with the r-axSpA subgroup (p = 0.004).

**TABLE 3:** Achievement of low disease activity (LDA) or very low disease activity (VLDA) at 6, 12 and 24 months in patient subgroups.

Timepoint	Lines of prior biologic therapy			Sex			Type of axSpA		
	≤2	>2	P-value	Female	Male	P-value	Nr-axSpA	r-axSpA	P-value
LDA									
6 months	20/43 (46.5)	52/126 (41.3)	0.548	42/91 (46.2)	30/78 (38.5)	0.313	48/119 (40.3)	23/49 (46.9)	0.431
12 months	23/34 (67.6)	52/88 (59.1)	0.384	45/66 (68.2)	30/56 (53.6)	0.098	55/90 (61.1)	19/31 (61.3)	0.986
24 months	15/16 (93.8)	36/46 (78.3)	0.261	32/37 (86.5)	19/25 (76.0)	0.326	***42/46 (91***. ***3)***	***9/16 (56***. ***3)***	***0***. ***004***
VLDA									
6 months	7/43 (16.3)	18/126 (14.3)	0.751	15/91 (16.5)	10/78 (12.8)	0.504	15/119 (12.6)	10/49 (20.4)	0.196
12 months	13/33 (39.4)	26/88 (19.5)	0.302	***27/65 (41***. ***5)***	***12/56 (21***. ***4)***	***0***. ***018***	29/90 (32.2)	9/30 (30.0)	0.821
24 months	11/16 (63.8)	22/46 (47.8)	0.261	20/37 (54.1)	13/25 (52.0)	0.874	26/46 (56.5)	7/16 (43.8)	0.378

Statistically significant differences are shown in *bold italics*.

### Drug retention rate

The DRR for upadacitinib was 91.9% (170/185) at 6 months, 79.2% (114/144) at 12 months and 55.4% (46/83) at 24 months. There were generally no significant differences in DRR at 6, 12 and 24 months between subgroups of patients (Supplementary Table S4), except that 6-month DRR was significantly greater in patients with disease duration ≤5 years versus >5 years (100% vs. 89.1%; p = 0.043). The 24-month DRR was greater in HLA-B27-negative patients compared with HLA-B27-positive patients (59.7% vs. 33.3%), with borderline statistical significance (p = 0.054). For the most part, there were no differences in DRR in subgroups of patients with specific comorbidities (Supplementary Table S5), except that DRR at 12 months was significantly lower in patients with versus without diabetes (42.9% vs. 80.3%; p = 0.039).

### Predictors of DRR

In the univariate analysis, BASDAI score at baseline was the only variable significantly associated with DRR at 6 months or upadacitinib interruption (Table 4). Patients with a baseline BASDAI indicative of LDA (≤4) had a more than tenfold chance of still being on upadacitinib at 6 months (OR 10.222 [95% CI 2.878–32.312]; p < 0.0001). The higher the baseline BASDAI score, the greater the chance of upadacitinib discontinuation and the lower the chance of drug retention at 6 months (Table 4).

**TABLE 4:** Univariate analysis of significant predictors of upadacitinib interruption or drug retention rate (DRR) at month 6.

Variable	DRR at 6 months		Upadacitinib interruption	
	Or (95% CI)	P-value	HR (95% CI)	P-value
Age at onset of AxSpA symptoms	1.007 (0.982–1.033)	0.580	1.008 (0.982–1.034)	0.561
Sex (female vs. male)	1.371 (0.742–2.536)	0.314	1.288 (0.669–2.479)	0.449
HLA-B27 status (positive vs. negative)	1.261 (0.586–2.710)	0.553	1.344 (0.631–2.863)	0.444
Sacroiliitis on MRI (negative vs. positive)	2.155 (0.777–5.987)	0.140	1.014 (0.391–2.624)	0.978
CRP level at baseline	1.003 (0.973–1.034)	0.863	1.008 (0.980–1.037)	0.586
**BASDAI at baseline**	***0***. ***594 (0***. ***476–0***. ***742)***	***<0***. ***0001***	***1***. ***325 (1***. ***062–1***. ***655)***	***0***. ***013***
Smoking (yes vs. no)	1.657 (0.791–3.473)	0.181	1.070 (0.515–2.223)	0.856
Previous lines of biologic therapy (≤2 vs. >2)	1.237 (0.617–2.483)	0.549	1.827 (0.759–4.397)	0.178

Statistically significant associations are shown in *bold italics.*

AxSpA, axial spondyloarthritis; BASDAI, bath ankylosing spondylitis disease activity index; CI, confidence interval; CRP, C-reactive protein; DRR, drug retention rate; HLA, human leukocyte antigen; MRI, magnetic resonance imaging; OR, odds ratio.

### NSAID and GC use

Matched data on NSAIDs use at baseline and 6 months was available in 132 patients, at 6 and 12 months in 95 patients and at 12 and 24 months in 56 patients. At baseline, 70 patients were taking NSAIDs (53.0%), dropping to 48 (36.4%) at 6 months (p < 0.0001). There were further reductions between 6 and 12 months, from 30/95 (31.6%) to 22/95 (23.2%), and between 12 and 24 months, from 10/56 (17.9%) to 8/56 (14.3%), but these changes were not statistically significant (p = 0.077 and 0.688, respectively). Similar significant changes in GCs use were seen in the first 6 months, from 21/151 (13.9%) to 10/151 (6.6%) (p = 0.003), but not between months 6 and 12 (6.4%–5.5%; p = 0.999) or 12 and 24 (7.1%–8.9%; p = 0.999).

### Safety

Overall, infections developed in 22/203 patients (10.8%) (Table 5); 12/22 patients (5.9%) had a single infection and 10 (4.9%) had more than one. Among the infections, five were varicella zoster virus (VZV) and three were herpes simplex virus (HSV) reactivation. None of the patients with a history of tuberculosis or hepatitis B experienced a reactivation of these infections. These results are consistent with those reported in clinical studies, where serious infections and herpes zoster infections occurred in a small proportion of patients (approximately 3%–4% annually). Serious cases of herpes zoster were rare (around 0.1%), and no cases of opportunistic infection or active tuberculosis were observed (Baraliakos et al., 2024).

**TABLE 5:** Infections in patients receiving upadacitinib.

Infections, n (%)	Upadacitinib (n = 203)
Any infection	22 (10.8)
Number of infections	​
1	12 (5.9)
2	4 (2.0)
3	5 (2.5)
5	1 (0.5)
Hospitalized for infection	4 (2.0)
Types of infection	​
VZV infection	5^a^ (2.5)
Urinary tract infection	4^b^ (2.0)
Bronchitis	3^c^ (1.5)
Upper respiratory tract infection	3^d^ (1.5)
Reactivation of HSV	3^b^ (1.5)
Diverticulitis	2^e^ (1.0)
Pulmonitis	1 (0.5)
COVID	1 (0.5)
Tonsillitis	1 (0.5)
Dental abscess	1 (0.5)
Urinary sepsis	1 (0.5)

^a^ Occurred with UTI, in one patient and with reactivation of HSV, in one patient.

^b^ occurred in one patient with VZV, infection.

^c^ one patient also had two episodes of pharyngitis and two episodes of fever.

^d^ one patient with pharyngitis also had bronchitis.

^e^ perforated in one patient.

COVID, coronavirus disease; HSV, herpes simplex virus; VZV, varicella zoster virus.

Six patients interrupted treatment and four were hospitalized because of infections (two for diverticulitis—one of which was perforated—one for a urinary tract infection and one for urinary sepsis). The patient with perforated diverticulitis required surgical intervention, antibiotics and discontinuation of upadacitinib.

Three patients were diagnosed with cancer during upadacitinib treatment, two with melanoma (one melanoma *in situ*) and one with chronic lymphocytic leukemia.

## Discussion

The current study demonstrates the effectiveness of upadacitinib in a real-world Italian cohort of patients with axSpA. Published data on the real-world effectiveness and safety of upadacitinib in axSpA patients is scarce, and to the best of our knowledge, this is the first study to investigate the DRR of upadacitinib in Europe. Although the results reflect a local, Italian, population, we believe that our experience may be relevant and could be extended to patients in other countries. Indeed, HLA-B27 negativity, in addition to the extensive prior treatment history, represents important characteristics of the Italian population that should be considered. Moreover, the male-to-female ratio in our cohort was 1:1, providing a more balanced and representative sample. We are also conscious that the following discussion is based on real-world experience, and variations may be expected compared with the highly selected populations enrolled in clinical trials.

We found that upadacitinib provides rapid and sustained effectiveness for up to 24 months in Italian patients with axSpA, as reflected by improved disease activity scores and indices (e.g., ASDAS and BASDAI), corroborating data from previous RCTs and meta-analyses involving upadacitinib (Baraliakos et al., 2024; Deodhar et al., 2022; van der Heijde et al., 2019; Ali et al., 2024; Tang et al., 2024; Yao et al., 2025; Navarro-Compan et al., 2025; Van den Bosch et al., 2025). Measures of disease activity are core requirements for the assessment of treatment effectiveness in axSpA (Navarro-Compan et al., 2021), and BASDAI and ASDAS are indices commonly used to assess disease activity in clinical practice and RCTs (Kumthekar et al., 2024).

The proportion of patients achieving ASDAS-LDA or ASDAS-VLDA at 6, 12, and 24 months in our study compared favorably to the rates reported in the SELECT-AXIS 2 study (Deodhar et al., 2022; Van den Bosch et al., 2025). Similarly, the 6-month LDA and VLDA rates in our study were consistent with reports from a single-center real-world observational study among patients with spondyloarthritis receiving JAKis in Spain (Ramirez Huaranga et al., 2024). The LDA and VLDA rates were particularly notable in patients nr-axSpA compared with r-axSpA, at all timepoints (Van den Bosch et al., 2025). Recent data from the SELECT AXIS 1 and 2 studies indicate that, irrespective of axSpA subtype, over 85% of patients who achieve early response (measured by disease activity) maintain that response over 2 years of treatment, which is consistent with our findings of sustained efficacy associated with prolonged treatment with upadacitinib.

Although axSpA subtype was a significant determinant of LDA at 24 months in our study, female sex was a significant determinant of VLDA at 12 months, with a significantly higher rate of VLDA vs. males (41.5% vs. 21.4%). This contrasts with the data on TNF or IL-17 inhibitors, wherein males have better rates of LDA or VLDA vs. females (Ga et al., 2025). Sex was not a determinant of treatment response in a real-world analysis of JAKis conducted in Spain (Ramirez Huaranga et al., 2024) in which baseline disease activity was found to be the only significant predictor of treatment response (Ramirez Huaranga et al., 2024). Potential sex differences in outcome parameters with upadacitinib warrant further investigation.

Additionally, the significant reduction in NSAIDs use observed at 6 months–from 53% at baseline to 36.4% (p < 0.0001) – may reflect the early clinical effectiveness of upadacitinib in controlling symptoms, consistent with previous reports of early pain control in real-world patients undergoing upadacitinib (Poddubnyy et al., 2025). This decline suggests that upadacitinib may help reduce the need for concomitant symptomatic therapies such as NSAIDs, potentially improving the overall safety profile and reducing treatment burden for patients with axSpA. A significant similar reduction was observed in GC use during the first 6 months of treatment, decreasing from 13.9% to 6.6% (p = 0.003). This finding may further support the early anti-inflammatory efficacy of upadacitinib, potentially reducing the need for adjunctive corticosteroid therapy and thereby lowering the risk of long-term GC-related AEs.

We found that over 90% of patients who start upadacitinib were still taking it at 6 months, and about 80% at 12 months, which is comparable to retention rates reported among Italian axSpA patients taking biologics/csDMARDs (Gentileschi et al., 2024; Currado et al., 2024; Christiansen et al., 2022), and Spanish patients with axSpA taking JAKis in the BIOBADASER Registry Phase III (Hernandez-Cruz et al., 2024). Importantly, data from the BIOBADASER 3.0 Registry indicates that 64% of patients with axSpA continued treatment with JAKis at 2 years (Van den Bosch et al., 2025), supporting the sustained use of this therapeutic class.

The efficacy and DRR of upadacitinib were evaluated in a real-world population characterized by high disease activity and a median of two prior biologic therapy failures. Our data suggests that disease activity at baseline may be a significant determinant of DRR of upadacitinib in real-world clinical practice. In our analysis, patients with a baseline BASDAI score ≤4 had a tenfold increased likelihood of continuing to take upadacitinib at 6 months vs. patients with baseline BASDAI >4. When evaluated as a continuous variable, each 1-point increment in baseline BASDAI score was associated with a 30% increase in the risk of interrupting treatment before month 6. The finding that higher disease activity may be a risk factor for earlier treatment discontinuation has been shown previously in patients with axSpA (Currado et al., 2024; Sakr et al., 2022), including Italian axSpA patients, irrespective of drug class. This likely reflects the complex interplay between disease activity and other symptoms (e.g., fatigue, pain), psychological comorbidities (e.g., anxiety, depression), quality of life, sleep disturbances, psychosocial factors and treatment satisfaction (Currado et al., 2024; Sakr et al., 2022; Kieskamp et al., 2025; Reddy et al., 2022; Webers et al., 2019). A *post hoc* analysis from the SELECT-AXIS 2 study showed that patients with baseline BASDAI ≥8 in at least 3 of 5 components or Maastricht AS Enthesitis Score ≥10, had significantly higher rates of anxiety and depression, pain and fatigue, and were less likely to report treatment as being effective, irrespective of whether they were receiving upadacitinib or placebo (Dougados et al., 2023).

The profile of AEs in our study was generally consistent with the known safety profile of upadacitinib (Burmester et al., 2023; Burmester et al., 2024). In our study, 22 patients (10.8%) developed an infection, but only 10/22 patients developed more than one infection during the course of upadacitinib treatment. Most of these infections were not serious and only four cases required hospitalization. The risk of serious infections during treatment with JAKis is similar in magnitude to the risk with biologic DMARDs (Adas et al., 2022). Five patients in our cohort developed Zoster infections (2.5%), a rate consistent with previous reports (Burmester et al., 2023; Burmester et al., 2024).

One patient in our cohort developed perforated diverticulitis requiring surgery, during 12 months of follow-up. Gastrointestinal (GI) perforation has been reported before in patients receiving JAKis, with an estimated incidence of 2.1 per 1,000 person-years (Hoisnard et al., 2024). The risk appears to be similar irrespective of the JAKi used, but there is evidence that the risk may be increased in patients taking concomitant NSAIDs or oral GCs (Goldman et al., 2025). Further research is required to clarify the incidence and risk factors for GI perforation in patients taking JAKis (Mikaeili et al., 2025).

Three patients developed malignancies (including one melanoma *in situ*), none of whom had a prior cancer history. The incidence rate corroborates that observed in the general Italian population data and prior findings that spondyloarthritis patients may carry a higher intrinsic cancer risk, especially those with concomitant psoriasis or phototherapy (Rubbert-Roth et al., 2024; International Agency for Research on Cancer, 2022). Clinical trial data suggests a low intrinsic risk of malignancy with upadacitinib, with age being the main risk factor (Favalli et al., 2025; Palmieri et al., 2015).

Only 22.7% of patients in our cohort were positive for HLA-B27 and there was a notable difference in HLA-B27 positivity between patients in North Italy versus Central or Southern Italy. The rate of HLA-B27 positivity in our study was lower than in previous Italian studies (Chim et al., 2021; Lorenzin et al., 2023; Ramonda et al., 2024), which may reflect disease factors that affect the selection of upadacitinib as axSpA treatment. Patients who are HLA-B27 positive tend to have greater clinical response to TNF inhibitors compared to HLA-B27 negative patients (Akkoc et al., 2017). Our study population was probably enriched by patients with a poor response to TNF inhibitors, many of whom would be HLA-B27 negative. The regional difference in HLA-B27 positivity in our study group is consistent with the known North-South geographical gradient in HLA-B27 status, with higher rates of positivity in Northern Europe compared with the Mediterranean regions (Mathieu et al., 2009).

Among the strengths of our study is the collection of real-world evidence, to complement data from RCTs, providing invaluable insights into the effectiveness and safety of upadacitinib in a heterogeneous Italian population of SpA patients seen in daily clinical practice, who often differ from clinical trial populations (Jones et al., 2020). Thus, it is of the utmost importance to confirm that the real-world effectiveness and tolerability of treatments are consistent with data from RCTs. Although there is scarce real-world evidence on upadacitinib to date, our study enrolled a large cohort of patients. Moreover, compared to recent interim results from the UPSTAND observational study, which reported that 35.3% of axSpA patients achieved ASDAS–LDA at week 12% and 46.2% at week 24, our real-world cohort achieved substantially higher LDA rates at similar timepoints. Furthermore, although the UPSTAND interim analysis is yet to report long-term retention rates, the reported rate of discontinuations due to treatment emergent adverse events (16.7 events per 100 patient-years) suggests moderate persistence comparable to our 12-month DRR of nearly 80%, thus reinforcing the consistency of upadacitinib DRR in early real-world use (McCormick, 2025).

We would be remiss not to acknowledge the limitations of our study, including its observational and non-comparative design. In addition, reliance on univariate analyses precludes causal inference, and the potential influence of residual confounding cannot be excluded. Incomplete baseline data for some patients and decreasing availability of matched data over time may have further affected the robustness of longitudinal analyses. Furthermore, our findings may not be fully generalizable, as we enrolled a predominantly Italian, referral-based cohort. Nevertheless, these real-world data reflect routine clinical practice and underscore the need for larger, multicenter studies in more diverse populations to confirm and extend these results.

## Conclusion

This real-world Italian study demonstrated that upadacitinib has a high treatment retention rate of 79.2% at 12 months. Upadacitinib markedly improved disease activity indices, with higher rates of LDA and VLDA over time. Drug retention was consistent with prior biologic data, confirming its effectiveness in daily clinical practice. Safety and tolerability aligned with the known JAKis profile. These findings, alongside clinical trial evidence, reinforce ASAS/EULAR guidance supporting JAKis as effective options in axSpA patients with inadequate response to NSAIDs, establishing upadacitinib as a durable, effective, and well-tolerated treatment in real-world settings in this subset of patients (Ramiro et al., 2023). Larger, multicenter studies are needed to confirm and extend these results.

## Data Availability

The raw data supporting the conclusions of this article will be made available by the authors, without undue reservation.

## Members of the Spondyloarthritis and Psoriatic Arthritis SIR Study Group ‘Antonio Spadaro’

Alice Agostinelli (Department of Clinical and Molecular Sciences, University Hospital of the Marche Region, Ancona, Italy), Arianna D’Antonio (Rheumatology, Allergology and Clinical Immunology, Department of Systems Medicine, University of Rome Tor Vergata, Rome, Italy), Carla Gaggiano (Rheumatology Unit, Department of Medical Sciences, Surgery and Neuroscience, University of Siena, Siena University Hospital, Siena, Italy), Elena Fracassi (Rheumatology Unit, Department of Medicine, AOUI University of Verona, Verona, Italy), Gianluca Poncina (Rheumatology Unit, Department of Medicine DIMED, Padova University Hospital, Padova, Italy) Giorgio Amato (Division of Rheumatology, AOU Policlinico “G. Rodolico” San Marco, Catania, Italy) Giovanni Striani (Division of Rheumatology, Department of Medicine, University of Udine, Azienda Sanitaria Universitaria del Friuli Centrale, Udine, Italy) Giulia Marchionni (Department of Clinical and Molecular Sciences, University Hospital of the Marche Region, Ancona, Italy) Luca Ingrao (University of Milan, Milano, Italy) Nicoletta Luciano (Department of Biomedical Sciences, Humanitas University, Milan, Italy; Department of Rheumatology and Clinical Immunology, IRCCS Humanitas Research Hospital, Milan, Italy) Riccardo Terribili (Rheumatology Unit, Department of Medical Sciences, Surgery and Neuroscience, University of Siena, Siena University Hospital, Siena, Italy) Salvatore D’Angelo (Rheumatology Department of Lucania, San Carlo Hospital of Potenza, Potenza, Italy; Department of Health Sciences, University of Basilicata, Potenza, Italy) Valentina Cateni (Rheumatology Unit, Department of Medicine, AOUI University of Verona, Verona, Italy).
