# Supplementary material for: Real-world clinical experience with upadacitinib in a cohort of Italian patients with axial spondyloarthritis
Source: Front Pharmacol. 2026 Apr 29;17:1757110. doi: 10.3389/fphar.2026.1757110 (PMC13168771; doi:10.3389/fphar.2026.1757110)

# Supplementary material

##

## Supplementary Table S1. Comorbidities present at baseline

| **Comorbidity** | **Upadacitinib (n=203)** |
| --- | --- |
| Hypertension | 45/195 (22.2) |
| Cardiovascular disease^a^ | 4/152 (2.0) |
| Dyslipidemia | 45/197 (23.2) |
| Diabetes | 7/196 (3.4) |
| Metabolic syndrome | 11/152 (5.4) |
| Hyperuricemia | 5/197 (2.5) |
| Pulmonary disease^b^ | 4/197 (2.0) |
| Gastrointestinal disease^c^ | 12/197 (5.9) |
| Liver disease^d^ | 8/197 (3.9) |
| Thyroid dysfunction | 21/197 (10.3) |
| Osteoporosis | 17/197 (8.4) |
| Psychiatric condition^e^ | 15/197 (7.4) |
| Fibromyalgia | 49/197 (24.1) |
| Neurological disorders | 4/197 (2.0) |
| Chronic renal disease | 2/197 (1.0) |
| Neoplasia | 5/197 (2.5) |
| Prior tuberculosis infection^f^ | 9/198 (4.4) |
| Prior hepatitis B infection^g^ | 8/198 (3.9) |

^a^Includes acute myocardial infarction, stroke and other cardiovascular disease; ^b^Includes asthma and chronic obstructive pulmonary disease; ^c^Includes ulcer; ^d^Includes steatosis; ^e^Includes depression or psychosis; ^f^Positive Quantiferon test result; ^g^Positive for hepatitis B surface antigen.

## Supplementary Table S2. Characteristics of patients in the evaluable population (at least one evaluable factor at 24 months) and those not in this population.

| **Variable, n (%)** | **Evaluable population (n=63)^a^** | **Non-evaluable population (n=140)^a^** | **P-value** |
| --- | --- | --- | --- |
| Previous lines of therapy |  |  |  |
| ≤2 | 16 (25.4) | 35 (25.0) | 0.952 |
| >2 | 47 (74.6) | 105 (75.0) |  |
| Biologic naïve | 1/62 (1.6) | 12 (8.6) | 0.069 |
| Sex |  |  |  |
| Female | 38 (60.3) | 68 (48.6) | 0.121 |
| Male | 25 (39.7) | 72 (51.4) |  |
| Extra-articular manifestations |  |  |  |
| Psoriasis |  |  |  |
| Nail involvement | 11/61 (18.0) | 14/138 (10.1) | 0.122 |
| Peripheral arthritis | 51/61 (83.6) | 85 (60.7) | 0.001 |
| Enthesitis | 36/59 (61.0) | 60/138 (43.5) | 0.024 |
| Dactylitis | 7/61 (11.5) | 15/140 (10.7) | 0.874 |
| IBD | 15 (23.8) | 24 (17.1) | 0.265 |
| Uveitis | 1/61 (1.6) | 13 (9.3) | 0.050 |
| Family history^b^ | 14/61 (23.0) | 35 (25.0) | 0.756 |
| Comorbidities |  |  |  |
| Hypertension | 19/57 (33.3) | 26/138 (18.8) | 0.029 |
| Cardiovascular disease^c^ | 4/52 (7.7) | 0/100 (0) | 0.005 |
| Dyslipidaemia | 18/59 (30.5) | 29/138 (21.0) | 0.152 |
| Diabetes | 2/59 (3.4) | 5/137 (3.6) | 0.928 |
| Metabolic syndrome | 2/52 (3.8) | 9/100 (9.0) | 0.333 |
| Hyperuricaemia | 1/59 (1.7) | 4/138 (2.9) | 0.999 |
| Pulmonary disease^d^ | 1/59 (1.7) | 3/138 (2.2) | 0.999 |
| Gastrointestinal disease^e^ | 3/59 (5.1) | 9/138 (6.5) | 0.999 |
| Liver disease^f^ | 1/59 (1.7) | 7/138 (5.1) | 0.440 |
| Thyroid dysfunction | 3/59 (5.1) | 18/138 (13.0) | 0.097 |
| Osteoporosis | 5/59 (8.5) | 12/138 (8.7) | 0.960 |
| Psychiatric condition^g^ | 5/59 (8.5) | 10/138 (7.2) | 0.774 |
| Fibromyalgia | 10/59 (16.9) | 39/138 (28.3) | 0.093 |
| Neurological disorders | 0/59 (0) | 4/138 (2.9) | 0.319 |
| Chronic renal disease | 0/59 (0) | 2/138 (1.4) | 0.999 |
| Neoplasia | 2/59 (3.4) | 3/138 (2.2) | 0.637 |
| Prior hepatitis B infection^h^ | 2/58 (3.4) | 6 (4.3) | 0.999 |

^a^Denominator unless otherwise stated; ^b^family history for any inflammatory arthritis; ^c^Includes acute myocardial infarction, stroke and other cardiovascular disease; ^d^Includes asthma and chronic obstructive pulmonary disease; ^e^Includes ulcer; ^f^Includes steatosis; ^g^Includes depression or psychosis; ^h^Positive for hepatitis B surface antigen.

IBD, inflammatory bowel disease.

## Supplementary Table S3. Effectiveness parameters over time in the effectiveness population.

|  | **Baseline** | | **6 months** | | **12 months** | | **24 months** | |
| --- | --- | --- | --- | --- | --- | --- | --- | --- |
| **Parameter** | **N** | **Median (range)** | **N** | **Median (range)** | **N** | **Median (range)** | **N** | **Median (range)** |
| VAS-pain score | 197 | 8 (2–10) | 170 | 5 (0–10) | 131 | 4 (0–10) | 63 | 3 (0–9) |
| VAS-GH score | 155 | 7 (2–10) | 130 | 5 (0–10) | 96 | 4 (0–9) | 56 | 2.5 (0–9) |
| VAS-PA score | 157 | 7 (1–10) | 132 | 4 (0–10) | 97 | 2 (0–8) | 56 | 1 (0–8) |
| BASMI score | 82 | 0 (0–5) | 63 | 1 (0–4) | 36 | 0.5 (0–4) | 15 | 0 (0–2) |
| LEI score | 141 | 0.5 (0–6) | 120 | 0 (0–6) | 90 | 0 (0–4) | 51 | 0 (0–2) |
| HAQ score | 126 | 0.875 (0–7) | 108 | 0.5 (0–7) | 86 | 0.25 (0–8) | 52 | 0.25 (0–7) |
| BASDAI score | 188 | 5.9 (0–9.25) | 169 | 4.2 (0–9.0) | 130 | 2.85 (0–8.0) | 62 | 1.95 (0–6.4) |
| BASFI score | 121 | 5.3 (0–9.2) | 103 | 1.7 (0–7.5) | 72 | 1.8 (0–6.2) | 25 | 1.0 (0–5.0) |
| ASDAS score | 194 | 2.8 (0.9–5.0) | 171 | 2.0 (0.3–4.6) | 128 | 1.545 (0–4.0) | 63 | 1.1 (0–3.3) |
| ESR, mm/h | 184 | 11 (0–96) | 154 | 8 (0–66) | 121 | 7 (0–54) | 59 | 6 (0–56) |
| CRP, mg/dL | 197 | 1.0 (1.0–59.7) | 171 | 1.7 (0–50.0) | 130 | 1.0 (1.0–25.0) | 63 | 0.5 (0–7.4) |

ASDAS, Ankylosing Spondylitis Disease Activity Score; BASDAI, Bath Ankylosing Spondylitis Disease Activity Index; BASFI, Bath Ankylosing Spondylitis Functional Index; BASMI, Bath Ankylosing Spondylitis Metrology Index; CRP, C-reactive protein; ESR, erythrocyte sedimentation rate; GH, global health; HAQ, Health Assessment Questionnaire; LEI, Leeds Enthesitis Index; PA, physician’s assessment; VAS, visual analogue scale.

Supplementary Table S4. Drug retention rate (DRR) at 6, 12 and 24 months in patient subgroups based on demographic and clinical characteristics.

Statistically significant p values are shown in ***bold italics*.**

| Patient subgroups | 6 months | | | 12 months | | | 24 months | | |
| --- | --- | --- | --- | --- | --- | --- | --- | --- | --- |
|  | **n** | **DRR** | **p value** | **n** | **DRR** | **p value** | **n** | **DRR** | **p value** |
| Prior lines of biologic therapy |  |  |  |  |  |  |  |  |  |
| ≤2 | 47 | 95.7 | 0.999 | 35 | 88.6 | 0.495 | 19 | 68.4 | 0.458 |
| >2 | 54 | 94.4 |  | 35 | 82.9 |  | 16 | 56.3 |  |
| Sex |  |  |  |  |  |  |  |  |  |
| Female | 97 | 91.8 | 0.942 | 75 | 81.3 | 0.504 | 46 | 60.9 | 0.266 |
| Male | 88 | 92.0 |  | 69 | 76.8 |  | 37 | 48.6 |  |
| Subtype of axSpA |  |  |  |  |  |  |  |  |  |
| Non-radiographic | 133 | 91.0 | 0.764 | 104 | 77.9 | 0.586 | 66 | 54.5 | 0.752 |
| Radiographic | 51 | 94.1 |  | 39 | 82.1 |  | 17 | 58.8 |  |
| Duration of disease |  |  |  |  |  |  |  |  |  |
| ≤5 years | 38 | 100.0 | ***0***.***043*** | 25 | 84.0 | 0.465 | 11 | 45.5 | 0.581 |
| >5 years | 138 | 89.1 |  | 115 | 77.4 |  | 68 | 54.4 |  |
| Age |  |  |  |  |  |  |  |  |  |
| ≤50 years | 75 | 89.3 | 0.292 | 56 | 76.8 | 0.575 | 26 | 42.3 | 0.105 |
| >50 years | 110 | 93.6 |  | 88 | 80.7 |  | 57 | 61.4 |  |
| HLA-B27 status |  |  |  |  |  |  |  |  |  |
| Positive | 41 | 85.4 | 0.112 | 30 | 73.3 | 0.389 | 15 | 33.3 | 0.054 |
| Negative | 139 | 93.5 |  | 113 | 80.5 |  | 67 | 59.7 |  |
| Smoking status |  |  |  |  |  |  |  |  |  |
| Yes | 42 | 90.5 | 0.754 | 40 | 85.0 | 0.263 | 22 | 59.1 | 0.599 |
| No | 137 | 92.0 |  | 102 | 76.5 |  | 59 | 52.5 |  |
| Sacroiliitis on MRI |  |  |  |  |  |  |  |  |  |
| Present | 159 | 91.8 | 0.999 | 118 | 77.3 | 0.779 | 64 | 53.1 | 0.393 |
| Absent | 22 | 90.9 |  | 22 | 79.7 |  | 17 | 64.7 |  |
| Peripheral arthritis |  |  |  |  |  |  |  |  |  |
| Present | 125 | 92.8 | 0.564 | 98 | 79.6 | 0.855 | 62 | 58.1 | 0.405 |
| Absent | 58 | 89.7 |  | 46 | 78.3 |  | 21 | 47.6 |  |
| Enthesitis |  |  |  |  |  |  |  |  |  |
| Present | 85 | 91.8 | 0.947 | 68 | 75.0 | 0.297 | 51 | 60.8 | 0.217 |
| Absent | 94 | 91.5 |  | 72 | 83.3 |  | 30 | 46.7 |  |
| Dactylitis |  |  |  |  |  |  |  |  |  |
| Present | 20 | 90.0 | 0.671 | 17 | 82.4 | 0.999 | 9 | 66.7 | 0.725 |
| Absent | 163 | 92.0 |  | 127 | 78.7 |  | 74 | 54.1 |  |
| Intestinal involvement |  |  |  |  |  |  |  |  |  |
| Present | 35 | 91.4 | 0.999 | 30 | 76.7 | 0.705 | 12 | 41.5 | 0.300 |
| Absent | 138 | 92.0 |  | 114 | 79.8 |  | 71 | 57.7 |  |
| Uveitis |  |  |  |  |  |  |  |  |  |
| Present | 11 | 90.9 | 0.999 | 9 | 77.8 | 0.999 | 2 | 0 | 0.196 |
| Absent | 172 | 91.1 |  | 135 | 79.3 |  | 81 | 56.8 |  |

axSpA, axial spondyloarthritis; DRR, drug retention rate; HLA, human leukocyte antigen; MRI, magnetic resonance imaging.

**Supplementary Table S5. Drug retention rate (DRR) at 6, 12 and 24 months in patient subgroups based on the presence/absence of comorbidities.**

Statistically significant p values are shown in ***bold italics*.**

| **Patient subgroups** | **6 months** | | | **12 months** | | | **24 months** | | |
| --- | --- | --- | --- | --- | --- | --- | --- | --- | --- |
|  | **n** | **DRR** | **p value** | **n** | **DRR** | **p value** | **n** | **DRR** | **p value** |
| Hypertension |  |  |  |  |  |  |  |  |  |
| Present | 44 | 93.2 | 0.765 | 39 | 74.4 | 0.468 | 28 | 64.3 | 0.191 |
| Absent | 133 | 100.0 |  | 100 | 80.0 |  | 53 | 49.1 |  |
| Dyslipidaemia |  |  |  |  |  |  |  |  |  |
| Present | 45 | 91.1 | 0.999 | 39 | 76.9 | 0.789 | 30 | 51.0 | 0.431 |
| Absent | 134 | 91.8 |  | 100 | 79.0 |  | 51 | 60.0 |  |
| Cardiovascular disease^a^ |  |  |  |  |  |  |  |  |  |
| Present | 4 | 100.0 | 0.999 | 4 | 100.0 | 0.570 | 2 | 50.0 | 0.999 |
| Absent | 130 | 90.0 |  | 98 | 75.5 |  | 62 | 54.8 |  |
| Diabetes |  |  |  |  |  |  |  |  |  |
| Present | 7 | 71.4 | 0.108 | 7 | 42.9 | ***0***.***039*** | 7 | 42.9 | 0.697 |
| Absent | 172 | 92.4 |  | 132 | 80.3 |  | 74 | 55.4 |  |
| Hyperuricaemia |  |  |  |  |  |  |  |  |  |
| Present | 5 | 100.0 | 0.999 | 5 | 60.0 | 0.294 | 3 | 33.3 | 0.590 |
| Absent | 159 | 91.4 |  | 134 | 79.1 |  | 78 | 55.1 |  |
| Pulmonary disease^b^ |  |  |  |  |  |  |  |  |  |
| Present | 4 | 100.0 | 0.999 | 4 | 75.0 | 0.999 | 2 | 50.0 | 0.999 |
| Absent | 175 | 91.4 |  | 135 | 78.5 |  | 79 | 54.4 |  |
| Gastrointestinal disease^c^ |  |  |  |  |  |  |  |  |  |
| Present | 11 | 81.8 | 0.232 | 10 | 70.0 | 0.450 | 5 | 40.0 | 0.656 |
| Absent | 168 | 92.3 |  | 129 | 79.1 |  | 76 | 55.3 |  |
| Liver disease^d^ |  |  |  |  |  |  |  |  |  |
| Present | 7 | 71.4 | 0.108 | 5 | 40.0 | 0.067 | 4 | 25.0 | 0.327 |
| Absent | 172 | 92.4 |  | 134 | 79.9 |  | 77 | 55.8 |  |
| Metabolic syndrome |  |  |  |  |  |  |  |  |  |
| Present | 9 | 77.8 | 0.212 | 8 | 50.0 | 0.086 | 7 | 42.9 | 0.693 |
| Absent | 125 | 91.2 |  | 94 | 78.7 |  | 59 | 55.9 |  |
| Thyroid disease |  |  |  |  |  |  |  |  |  |
| Present | 19 | 84.2 | 0.202 | 11 | 63.6 | 0.251 | 8 | 37.5 | 0.459 |
| Absent | 160 | 92.5 |  | 128 | 79.7 |  | 73 | 56.2 |  |
| Osteoporosis |  |  |  |  |  |  |  |  |  |
| Present | 15 | 93.3 | 0.999 | 11 | 72.7 | 0.703 | 6 | 50.0 | 0.999 |
| Absent | 164 | 91.5 |  | 128 | 78.9 |  | 75 | 54.7 |  |
| Psychiatric disorders^e^ |  |  |  |  |  |  |  |  |  |
| Present | 14 | 100.0 | 0.611 | 11 | 72.7 | 0.703 | 8 | 50.0 | 0.999 |
| Absent | 165 | 90.9 |  | 128 | 78.9 |  | 73 | 54.8 |  |
| Fibromyalgia |  |  |  |  |  |  |  |  |  |
| Present | 46 | 93.5 | 0.763 | 35 | 74.3 | 0.485 | 21 | 52.4 | 0.999 |
| Absent | 133 | 91.0 |  | 104 | 79.8 |  | 60 | 55.0 |  |
| Neurological disease |  |  |  |  |  |  |  |  |  |
| Present | 4 | 100.0 | 0.999 | 4 | 50.0 | 0.203 | 2 | 0 | 0.206 |
| Absent | 175 | 91.4 |  | 135 | 79.3 |  | 79 | 55.7 |  |
| Chronic renal disease |  |  |  |  |  |  |  |  |  |
| Present | 2 | 100.0 | 0.999 | 1 | 100.0 | 0.999 | 0 | 0 | 0.999 |
| Absent | 177 | 91.5 |  | 138 | 78.3 |  | 81 | 54.3 |  |
| Cancer history |  |  |  |  |  |  |  |  |  |
| Present | 5 | 100.0 | 0.999 | 5 | 100.0 | 0.585 | 2 | 100.0 | 0.498 |
| Absent | 174 | 91.4 |  | 134 | 77.6 |  | 79 | 53.2 |  |

^a^Includes acute myocardial infarction, stroke and other cardiovascular disease; ^b^Includes asthma and chronic obstructive pulmonary disease; ^c^Includes ulcer; ^d^Includes steatosis; ^e^Includes depression or psychosis

## Supplementary Figure S1. Proportions of patients with low disease activity (LDA) or very low disease activity (VLDA) among patients with paired data each timepoint in the effectiveness population.

ASDAS, Ankylosing Spondylitis Disease Activity Score; BASDAI, Bath Ankylosing Spondylitis Disease Activity Index *p<0.05, ‡p=0.007, †p<0.0001 vs preceding timepoint. BASDAI-LDA is a score <4, BASDAI-VLDA is a score <2, ASDAS-LDA is a score <2.1, ASDAS-VLDA is a score <1.3.


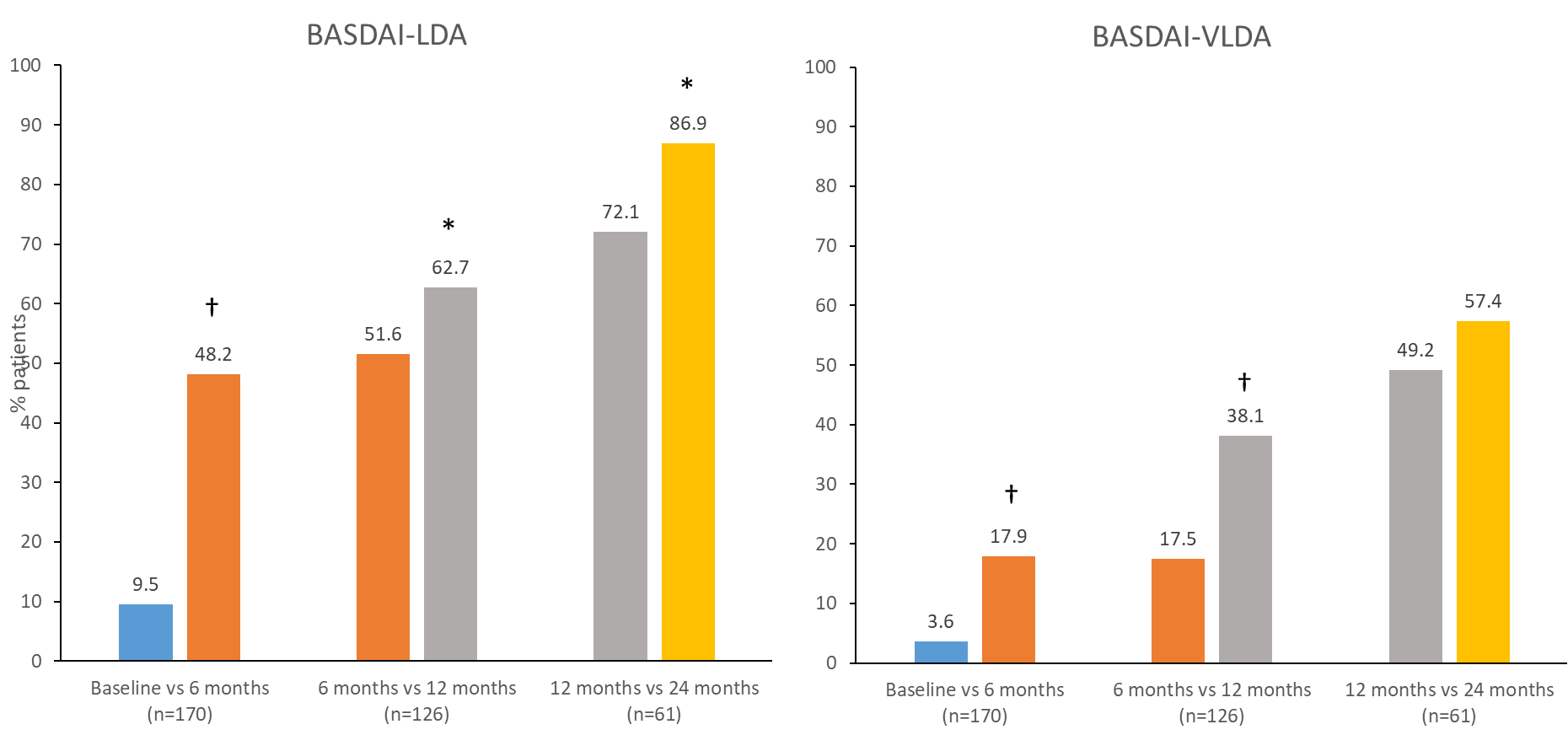


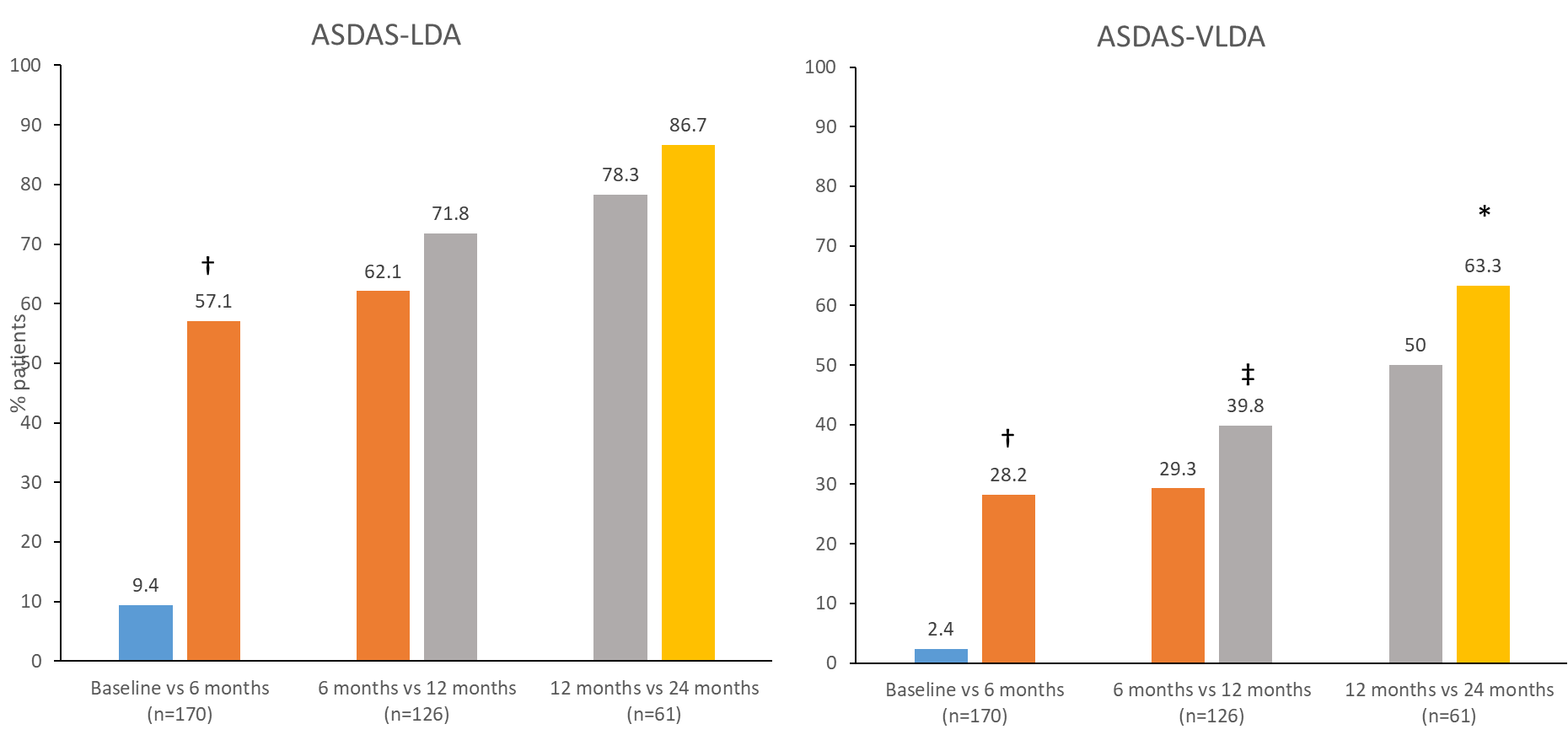

Supplement: Supplementary file 1 [file Supplementaryfile1.docx]
